# Bed Stability Control in Pulsed Fluidized-Bed Agglomeration of Instant Riceberry Powder Using an Image-Processing Technique

**DOI:** 10.3390/foods13121859

**Published:** 2024-06-13

**Authors:** Wasan Duangkhamchan, Prarin Chupawa, Naoshi Kondo, Donludee Jaisut

**Affiliations:** 1 Research Unit of Process Design and Automation, Faculty of Engineering, Mahasarakham University, Maha Sarakham 44150, Thailand; wasan.d@msu.ac.th; 2 Research Unit of Smart Process Design and Automation, Mahasarakham University, Maha Sarakham 44150, Thailand; prarin.c@msu.ac.th; 3 Research Unit of Mechatronics Engineering, Faculty of Engineering, Mahasarakham University, Maha Sarakham 44150, Thailand; 4 Division of Environmental Science and Technology, Graduate School of Agriculture, Kyoto University, Kyoto 606-8502, Japan; kondo.naoshi.6w@kyoto-u.ac.jp; 5 Department of Farm Mechanics, Faculty of Agriculture, Kasetsart University, Bangkok 10900, Thailand

**Keywords:** instant rice powder, image processing, pulsed fluid bed agglomeration, cohesive powder, instantaneous property, bed stability

## Abstract

The problematic cohesiveness of food powders can commonly be solved using pulsed fluidized-bed agglomeration. However, progressively larger granules may result in unstable fluidization. The aims of this research study were to investigate fluid bed expansion as affected by particle enlargement and to control its stability using an image-processing technique. Instant riceberry powder (IRP) was agglomerated using varied air pulsation frequencies (1, 2.5, and 4 Hz). Bed expansion captured by image processing revealed that expanded bed height decreased with agglomeration time. The results showed an enlargement of agglomerated IRP, expressed in D_10_, D_50_, and D_90_, with narrower distribution presented by span, and an improvement in bulk and reconstitution properties. The reduced Carr index (22–27%) and Hausner ratio (1.28–1.38) presented fair flowability and intermediate cohesiveness, respectively. Additionally, airflow during agglomerate growth was progressively adjusted using the image-processing method to enhance bed hydrodynamic stability, leading to improved process efficiency and product quality. This proposed approach has potential applications in the food powder manufacturing industry, particularly by enhancing the fluidization of cohesive particles with cracks and channels.

## 1. Introduction

Pigmented rice is rapidly gaining consumer preference over white rice due to its higher nutritional content and antioxidant activity. The consumption of pigmented rice has also been linked to a decreased risk of hyperlipidemia and diabetes [1]. However, brown and pigmented rice varieties have a coarse pericarp and seed coat, with longer cooking or rehydrating time for whole grains [2,3,4]. As a result, alternative products, such as instant beverage powders, have gained attention as convenience foods with high health benefits [2,4].

Instant pigmented rice powder has high dissolubility in hot water but is prone to problematic agglomeration during rehydration [5]. Starch in the powder gelatinizes at first contact with hot water and simultaneously forms a gel film wrapping the ungelatinized starch granule. As a result, hot water cannot penetrate this shell, leading to incomplete dissolution and some agglomeration of cohesive fine particles with poor flowability and low wettability. This problem can be solved by improving the wettability and bulk properties of the powder by means of fluidized-bed agglomeration, enlarging the fine particles, thereby increasing their porosity, wettability, and flowability [2].

Fluidized-bed agglomeration offers the transformative capability to convert particulate food substances into granular forms, thereby enhancing their instant properties. However, conventional food powder manufacturing processes, such as spray drying or milling, often result in the creation of cohesive particles with cracks and channels, posing challenges to their effective fluidization [6]. Recently, pulsed fluidized-bed agglomeration has been successfully used to improve bed homogeneity by introducing fluidizing air through a powder bed intermittently, resulting in reduced powder cohesiveness. Air pulsation reduces the minimum air velocity required for the fluidization regime in conventional processes, thereby minimizing the loss of fine particles due to elutriation [6]. The pulsed fluidized-bed agglomeration process involves spraying a binder onto solid particles intermittently fluidized by a hot air stream with a specific frequency. The collision of wetted particles results in larger and more porous granules, leading to improved instantaneous attributes [6,7,8].

The pulsed agglomeration process is influenced by several parameters, including pulsation frequency, fluidization air flow rate, binder feed rate, and binder attributes. Dacanal and Menegalli [7] conducted a study on instant soy protein isolate (SPI) using fluidized-bed agglomeration. They found that an air pulsation frequency of 10 Hz resulted in improved fluidization, increased particle diameter, and higher process yield, while Dacanal et al. [9] focused on spray-dried SPI and determined that pulsed agglomeration at 10 Hz led to size enlargement in raw SPI particles. Ali et al. [10] employed pulsed fluidizing air with a square wave frequency of 0.1 Hz, demonstrating a reduction in average agglomerate size for nano-powders. Dacanal et al. [6] explored the use of an air pulsation system at variable frequencies for cohesive starch particles, observing larger agglomerates with improved flowability and faster agglomeration rates. Nascimento et al. [11] used pulsed fluidized-bed agglomeration with varied air temperatures and binder flow rates for pea protein isolate (PPI), resulting in PPI agglomerates with better fluid dynamics behavior and dispersibility due to larger and more porous granules. Among the various pulsation frequencies evaluated by Nascimento et al. [12], 4 Hz air pulsation showed superior fluid dynamics behavior, characterized by lower fluidization velocity, larger granules, increased porosity, and shorter wetting time compared to raw PPI. However, the enlargement of particles during agglomeration may reduce bed expansion affecting bed hydrodynamics. Custodio et al. [13] increased the fluidizing air flow rate incrementally to maintain stable bed expansion and achieved a narrow particle distribution. Increasing the air flow rate compensated for particle size increase, sustaining a stable bed height and enabling larger particles to reach the wetting active zone, resulting in larger final agglomerates. Therefore, achieving optimal particle characteristics and instant properties requires considering both operating parameters and bed stability.

Despite extensive research on agglomeration parameters and their effects on the final product, limited studies have focused on enhancing the instant properties of starch-based powders, such as brown rice, through fluidized-bed agglomeration. The impact of bed expansion control on agglomerate characteristics has also been scarcely reported. Therefore, this study investigated the influence of pulsation frequency on the bed expansion of instant brown rice powder. Furthermore, an image-processing technique was employed to control bed stability, and finally, its impact on powder quality attributes was analyzed.

## 2. Materials and Methods

### 2.1. Materials and Sample Preparation

Riceberry (*Oryza sativa* L.) was chosen as the raw material due to its high anthocyanin content and antioxidant properties. A pregelatinized riceberry sample was prepared by cooking 150 g of raw riceberry with 450 mL of distilled water using a conventional domestic electric rice cooker and was then tray-dried at 60 °C until reaching the desired moisture content (10% wet basis). The dried rice grains were ground using a hammer mill (Lab Mill 120, PerkinElmer, Shelton, CT, USA) and the resulting instant riceberry powder (IRP) was sieved through an 80-mesh sieve, corresponding to a hole size of 177 μm.

### 2.2. Description of a Lab-Scale Fluidized-Bed Agglomeration System

**Figure 1:**
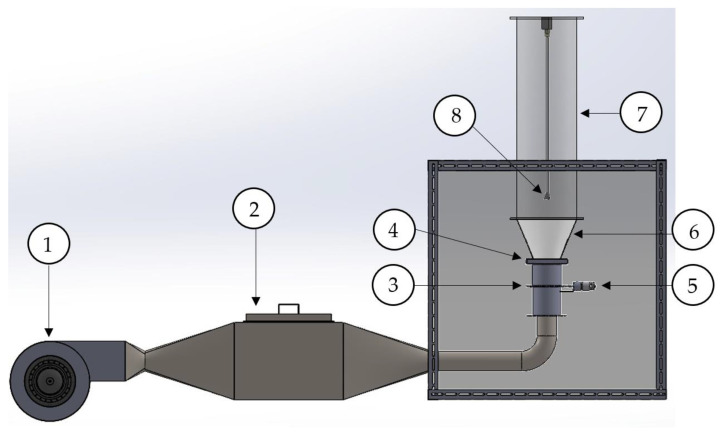
Schematic diagram of a lab-scale fluidized-bed agglomeration system.

The lab-scale fluidized-bed agglomeration apparatus, shown in Figure 1, consisted of an air blower connected to a three-phase 1 hp motor (Mitsubishi Electric Automation, Co., Ltd., Bangkok, Thailand) (number 1), an air heating system consisting of ten 1 kW finned heaters (number 2), a butterfly valve (number 3) connected to a 12-V DC motor (number 5), an air distributor (number 4), a plexiglass cone (number 6), and a cylindrical plexiglass chamber (number 7). Ambient air was supplied through a heating box with velocity and temperature controlled by an inverter (Model H-3200 Series, Haitec Transmission Equipment Co., Ltd., Hong Kong, China) and a PID (Proportional–Integral–Derivative, a control loop feedback mechanism) temperature controller (Model MAC-3D, Shimax Co., Ltd., Akita, Japan), respectively. Pulsed fluidizing air was supplied through a butterfly valve connected with a DC motor and air pulsation frequency was controlled via an L298N and Raspberry Pi 4B boards. A two-fluid nozzle (number 8) was situated at 0.3 m above an air distributor to provide top-spray fluidized-bed agglomeration.

### 2.3. Experimental Procedure

Two experimental scenarios were executed to explore the impact of pulsation frequency on bed expansion (in the first scenario) and to examine the influence of a bed control system on the fluidization behavior and quality of agglomerated IRP at varying air pulsation frequencies (in the second scenario). For all tests, a fixed quantity of the IRP sample weighing 300 g, which equates to a bed height of 0.045 m, was used. The minimum fluidization velocities (U_mf_) for each tested pulsation frequency were initially determined by analyzing the relationship between pressure drop and superficial air velocity, associated with visual inspection using a CCD camera (Model acA1300-200uc, Basler AG, Ahrensburg, Germany). For regular experiments, the fluidizing air velocity was set at 1.1 times the U_mf_, while for processes involving bed stability control, the air flow started at 1.1 U_mf_ and was then adjusted progressively. All agglomeration procedures were performed with a consistent pulsation frequency of 2.5 Hz and a fluidizing air temperature of 70 °C.

### 2.4. Analysis of Bed Expansion and Its Control

**Figure 2:**
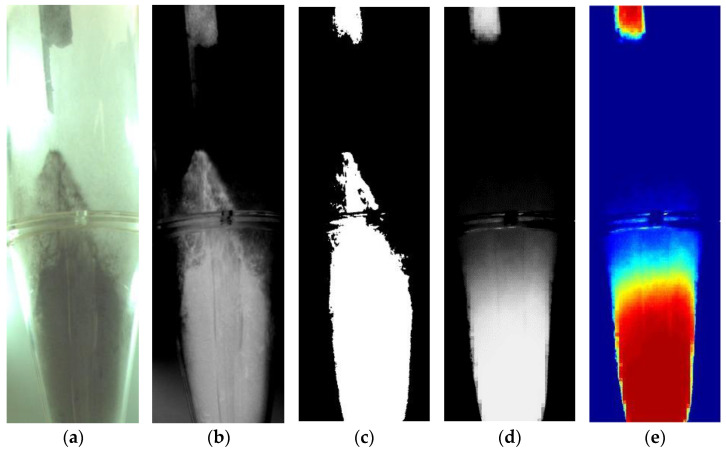
Image-processing procedure for evaluating bed height: (**a**) original image, (**b**) inverted gray-scale image, (**c**) converted binary image, (**d**) time-averaged image, (**e**) time-averaged contour.

The bed hydrodynamics behavior of IRP during pulsed fluidized-bed agglomeration was analyzed using an image-processing technique following Chupawa et al. [14] and Raut et al. [15]. Images (1280 × 1024 pixels in size) of an IRP bed during the agglomeration process were captured using a CCD camera (Model acA1300-200uc, Basler AG, Germany) at a speed of 30 frames per second. The original image was rotated 90 degrees clockwise, then cropped to a size of 280 × 886 pixels (Figure 2a), corresponding to a real area of 15.55 × 49.22 cm covering the height from bed bottom to nozzle opening. The images were first converted to grayscale format. Next, the images were sharpened, their colors were inverted (Figure 2b), and then they were converted to binary images (Figure 2c) using a threshold factor of 80. In the binary system, a value of 1 represented a solid-particle phase, while a value of 0 represented a fluidizing air phase. The resulting images were time-averaged (Figure 2d) and converted to a contour plot with values ranging from 0 (blue) to 1 (red), representing air and solid occupancy, respectively (Figure 2e).

The time-averaged image (Figure 2d) was divided into numerous horizontal bands, with a size of 280 × 18 pixels for each, corresponding to a height of 1 cm (49 sets of horizontal bands total), and solid occupancy (ϕ) was computed for each horizontal band using Equation (1), where P is the brightness value (0–255) in each pixel of the horizontal band set and n denotes the total number of pixels in the horizontal band set. Therefore, the sum of the solid-particle occupancy of every 18 rows averaged to the sum of the solid-particle occupancy of the actual heights, counted from the top (under the nozzle area) to the bottom of the dilution region (ϕ~1%), was considered the expanded bed height.

(1) $$\phi =\frac{\sum_{i=1}^{n}P_{i}}{n\times 255}$$

Bed hydrodynamics is generally unstable during the agglomeration process because enlarged and wetted granules affect the circulation and residence time in an active wetting zone [16]. In this study, the fluidizing air flow rate was progressively adjusted every 10 s using an image-processing method so that the expanded bed height remained stable. Bed height control was implemented using a combination of real-time image processing and feedback control. The fluidizing air flow rate was adjusted every 10 s, based on the processed image data. This adjustment was conducted automatically using a custom control algorithm implemented on a Raspberry Pi 4B board, which was interfaced with the CCD camera and the fluidizing air control system. The control system used the solid occupancy data from the processed images to determine the necessary adjustments to the flow rate. The total delay between image acquisition, processing, and flow rate adjustment was approximately 2–3 s, ensuring that bed height remained stable throughout the agglomeration process. While the primary control system operated in real time, a set of pre-collected images was used to train the control algorithm. Bed height control was tested using the fluidized bed agglomeration apparatus under a constant air temperature of 70 °C and 2.5 Hz air pulsation frequency for 30 min, while fluidizing air with a velocity of 1.1 U_mf_ was initially supplied to the system. The aforementioned CCD camera was used to capture the diluted region, specified as a solid occupancy less than 2.2%, at 30 cm above the chamber base with time intervals of 10 s. The fluidizing air velocity was subsequently adjusted to a set point of solid occupancy to provide steady bed hydrodynamics.

### 2.5. Characterization of Instant Riceberry Powder

IRP particle morphology was examined using a scanning electron microscope (SEM) (Tabletop Microscope, TM4000Plus, Hitachi, Hitachi High-Tech Ltd., Tokyo, Japan) operated at magnifications of 50× and 100× under a voltage of 15 kV.

The moisture content of the IRP sample was determined using a standard oven method. Three grams of the sample was dried in a hot air oven at 105 °C for 48 h, with results expressed as wet basis (wb) throughout this paper. Water activity (aw) representing the amount of water facilitating microorganism growth was measured using a water activity meter (Aqualab, Decagon, Washington, DC, USA).

The bulk density (ρ_b_) of IRP was determined using a graduated cylinder, and tapped density (ρ_tap_) was determined by manually tapping the cylinder 50 times. Particle density (ρ_p_) was measured in accordance with the method of Dacanal et al. [6] with minor modifications using the liquid displacement method using a glass pycnometer with n-heptane. The total porosity (ε) of IRP was computed using ρ_b_ and ρ_p_ values as expressed in Equation (2).

(2) $$\varepsilon =1-\frac{\rho_{b}}{\rho_{p}}$$

The flowability and cohesiveness of the powder were evaluated in terms of the Carr index (CI) and the Hausner ratio (HR), respectively, using the bulk (ρ_b_) and tapped (ρ_tap_) densities, as shown in Equations (3) and (4). The classifications of flowability and cohesiveness of IRP were as follows: CI < 15 (very good), 15 < CI < 20 (good), 20 < CI < 35 (fair), 35 < CI < 45 (bad), and CI > 45 (very bad); HR < 1.2 (low), 1.2 < HR < 1.4 (intermediate), and HR > 1.4 (high) [6].

(3) $$CI=\frac{\left(\rho_{\mathrm{tap}}-\rho_{b}\right)}{\rho_{\mathrm{tap}}}$$

(4) $$\mathrm{HR}=\frac{\rho_{\mathrm{tap}}}{\rho_{b}}$$

### 2.6. Particle Size and Distribution

Particle size and its distribution were determined using a laser scattering particle size distribution analyzer (Horiba LA-950V2, Horiba Ltd., Kyoto, Japan) associated with the wet analysis system using n-heptane (refractive index of 1.385) as dispersion medium. Laser diffraction assesses particle size based on the diameter of an equivalent volume sphere, resulting in volume-based particle size distributions, including D_50_, D_10_, and D_90_ values. The D_50_ percentile, also known as the mass median diameter or the median of the volume distribution, signifies that half of the sample particles are smaller and half are larger than this value. The D_10_ value means 10% of the sample particles are smaller than this measure, while the D_90_ value indicates 90% are smaller and 10% are larger. The distribution span, which measures the breadth of the distribution, was calculated as outlined in Equation (5) [13].

(5) $$\mathrm{span}=\frac{D_{90}-D_{10}}{D_{50}}$$

### 2.7. Reconstitution Properties

The reconstitution properties of IRP were evaluated by examining its wettability and dispersibility, using the analysis methods proposed by Jinapong et al. [17] with minor modifications. Wettability was defined as the time taken for the powder to completely sink in distilled water, while dispersibility referred to the ratio of dissolved powder to the total amount of power on a dry basis.

To determine wettability, 0.1 g of IRP (or agglomerates) was placed around a test tube positioned on a glass funnel in such a way that the powder sample was prevented from flowing through the lower funnel opening. The distance between the funnel bottom and water surface was maintained at 10 cm. The time interval between lifting the test tube and the point at which the powder became completely wetted was recorded as the wettability value.

For the dispersibility analysis procedure, 1 g of powder sample was dissolved in 10 mL of distilled water. After stirring for 15 s, reconstituted IRP was poured through a 70-mesh sieve with a perforate size of 210 μm. Supernatant liquid (1 mL) was transferred to a pan and subsequently dried under 105 ± 1 °C in a hot air oven for 4 h, with IRP dispersibility calculated using Equation (6).

(6) $$\%\mathrm{dispersibility}=\frac{\left(10+a\right)\times \%\mathrm{TS}}{a\times \frac{100-b}{100}}$$

where a denotes the powder mass used (g), b represents its moisture content (%wb), and %TS is dry matter of the reconstituted IRP after passing through the sieve.

### 2.8. Statistical Analysis

Mean values and standard deviations were used to express all the obtained values in this study, with data subjected to one-way analysis of variance (ANOVA). Duncan’s post hoc test was employed to determine difference between means, with significance level set at 95% (*p* < 0.05).

## 3. Results

### 3.1. Effect of Air Pulsatin Frequency on Bed Expansion

**Figure 3:**
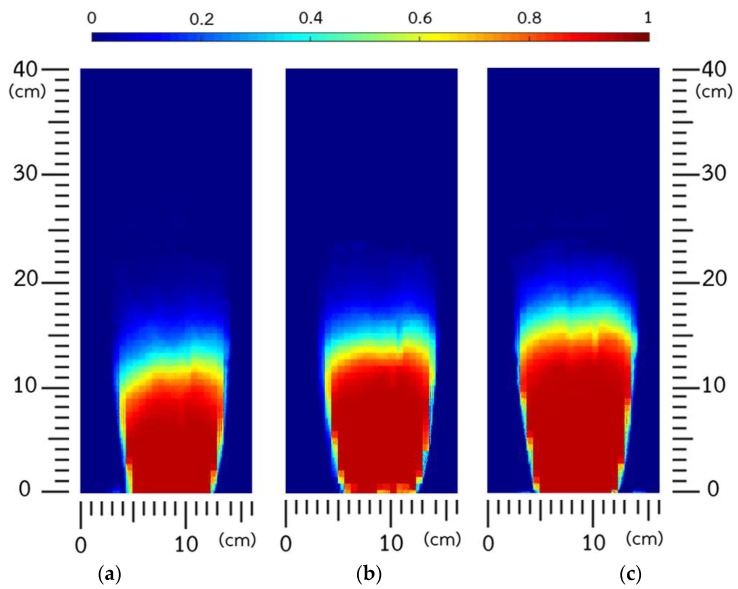
Time-averaged (10 min) solid occupancy at pulsation frequencies of (**a**) 1 Hz, (**b**) 2.5 Hz, and (**c**) 4 Hz.

Before analyzing how bed stability was affected by fluidized-bed agglomeration, the image-processing method was used to examine bed expansion under a constant fluidizing air flow rate with varied pulsation frequencies. Figure 3 shows contour plots of time-averaged solid occupancy, excluding the binder spray.

The visual results in Figure 3 indicated that the bed expansion of IRP increased with higher air pulsation frequency, resulting from greater fluidization homogeneity. At higher frequencies, the pulsed air flow was close to continuous fluidization with a lower amplitude of pulsation, resulting in decreased interparticle collision and subsequently reduced bed cohesiveness. More stable pulsation with sufficient time to facilitate particle movement resulted in improved fluidizability, with more effective particle trajectories into the wetting zone under uniform circulation during fluidized-bed agglomeration [6,13,16].

### 3.2. Bed Expansion as Affected by Agglomeration

#### 3.2.1. Solid Occupancy without Bed Height Control

**Figure 4:**
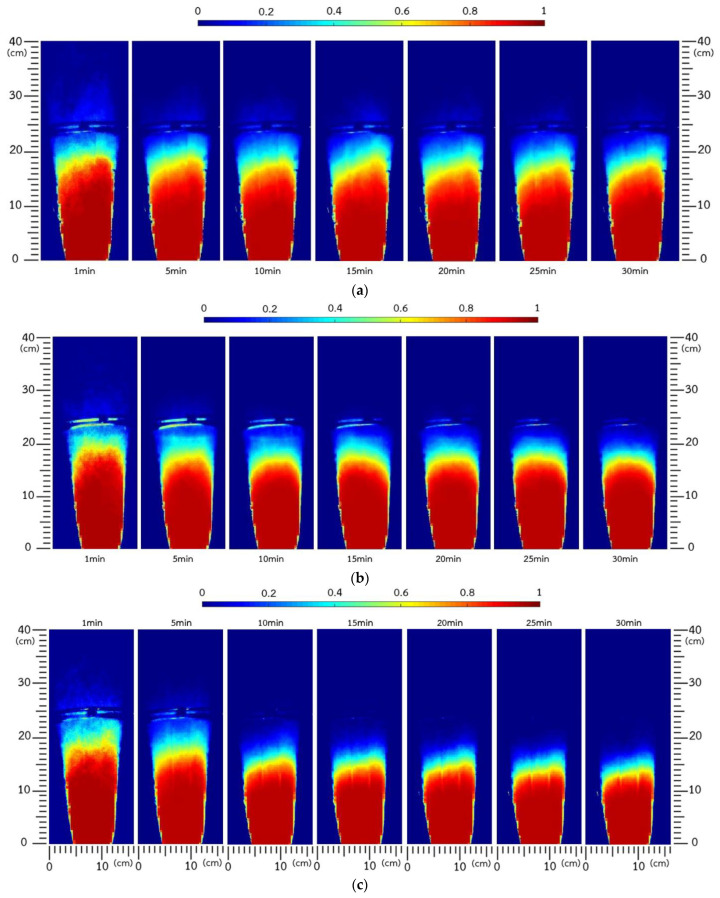
Time-averaged solid occupancy at pulsation frequencies of (**a**) 1 Hz, (**b**) 2.5 Hz, and (**c**) 4 Hz without bed height control.

In a top-spray fluidized-bed agglomeration process, particles are enlarged with progressive granule growth, leading to decreasing bed height [16]. Figure 4 shows contour plots of the solid occupancy time-averaged at 5 min intervals under agglomeration with different pulsation frequencies. The height of bed expansion visually decreased with time due to heavier enlarged granules under a constant flow rate of fluidizing air, which may affect the characteristics of the final agglomerates. Heavier and larger IRP granules moved upward with more gravitational force, resulting in shorter trajectories and consequently lower expanded bed height. Turchiuli et al. [16] also stated that bed hydrodynamics decreased with time due to particle enlargement as the agglomeration progressed. Our findings were consistent with those reported for many raw materials, such as rice protein concentrate [2], pea protein isolate [11,12], plant protein powder [13], and mushroom powder [18].

**Figure 5:**
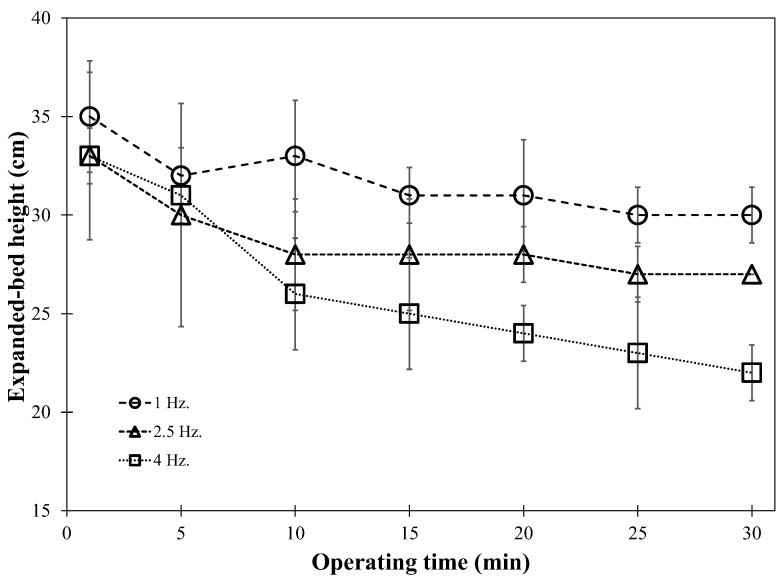
Expanded bed height as a function of operating time without bed stability control.

Due to the cohesion behavior of IRP, air pulsation was introduced into the fluidized bed to obtain better bed hydrodynamics. Figure 4a–c demonstrates time-averaged solid occupancy using pulsation frequencies of 1, 2.5, and 4 Hz, respectively. At each pulsation frequency, expanded bed height visually decreased with agglomeration time. The change in fluidized-bed behavior was more prominent when using a higher pulsation frequency, especially at 4 Hz (see Figure 4c). Higher air pulsation frequency increased stability, with sufficient time for particle movement, resulting in more effective entry into the active wetting zone, thereby facilitating particle enlargement. The influence of air pulsation frequency on changes in bed height was qualitatively confirmed by the profiles shown in Figure 5. The IRP bed expanded to a maximum height of 0.33–0.35 m at the beginning of the process, and then linearly reduced to 0.31, 0.28, and 0.23 m after agglomeration for 30 min at frequencies of 1, 2.5, and 4 Hz, respectively. The formation of larger granules also resulted in reduced bed expansion when using higher pulsation frequency. At higher frequencies, channeling formation was more intensively disrupted by the pulsed air stream, and the cohesive IRP was more easily fluidized at a higher velocity in the wetting zone and coalesced with the binder droplets forming the agglomerates. Dacanal et al. [6] also reported that using a higher pulsation frequency with greater disruption in channel formation provided better bed fluid hydrodynamics and, consequently, agglomeration efficiency. Nevertheless, extremely high frequencies of air pulsation exceeding the appropriate level may cause intense particle agitation, resulting in the granules breaking into fine powder due to a higher number of collisions, consequently reducing the agglomeration rate.

#### 3.2.2. Bed Expansion Controlled Using an Image-Processing Technique

**Figure 6:**
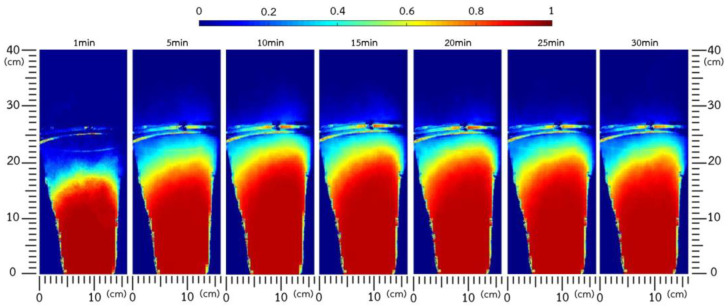
Time-averaged solid occupancy at pulsation frequencies of 4 Hz obtained using bed height control.

**Figure 7:**
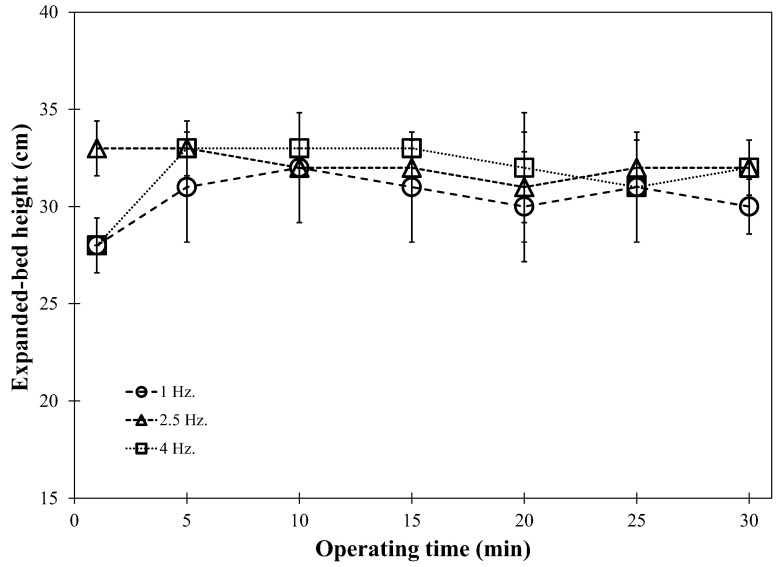
Comparison of expanded bed height profiles with bed height control at different air pulsation frequencies.

Once the IRP particles entered the active wetting zone situated just under the binder spray nozzle tip, the wetted particles collided with each other, forming agglomerates with larger sizes. Therefore, bed height and the velocity of particles decreased as the agglomeration process progressed (Figure 6), resulting in lower bed expansion and less occupancy in the binder spray zone. The main aim of this study was to enhance the stability of bed hydrodynamics by progressively adjusting the airflow during agglomeration growth using the image-processing method, enabling even larger particles to penetrate the active spray zone. Figure 6 only demonstrates the change in IRP bed expansion during the agglomeration process at an air pulsation frequency of 4 Hz, due to the similar manner observed among all frequencies tested. The IRP bed expansion was stable, achieving a height of about 0.30 m throughout the process. Bed stability during agglomeration was confirmed by the levelled-off profiles of the solid occupancy of the IRP sample, as shown in Figure 7. Custodio et al. [13] also reported that fluidization stability was maintained when increasing the inlet air flow rate.

Bed stability control is infrequently utilized in fluidized-bed agglomeration processes. Typically, the optimal air flow rate is determined based on the final qualities of the powder. Some studies have used manual air flow adjustments at fixed intervals. For example, Custodio et al. [13] gradually increased the fluidizing air flow rate, starting at 5 m^3^/h and incrementing it every 10 min until the process end. The proposed image-processing-based method offers significant improvements by providing real-time feedback. This technique captures and analyzes images of the fluidized bed, allowing for dynamic and automatic adjustments of the air flow rate to maintain stable bed expansion. Additionally, an advantage of the proposed method is that it controls bed height to the desired level, especially in the active wetting zone, thereby enhancing agglomeration performance. The scalability of this technique for industrial applications can be achieved by modifying the agglomeration chamber with plexiglass to capture a portion of the particle bed using a camera. Additionally, flow rates as a function of process time can be determined using lab-scale equipment and empirical data to scale up the main process parameters.

### 3.3. Quality Attributes

The quality attributes of the raw IRP were compared with those obtained by pulsed fluidized-bed agglomeration at a fixed pulsation frequency of 2.5 Hz and an initial inlet air velocity of 1.1 U_mf_ with and without bed control.

#### 3.3.1. Particle Morphology (SEM)

**Figure 8:**
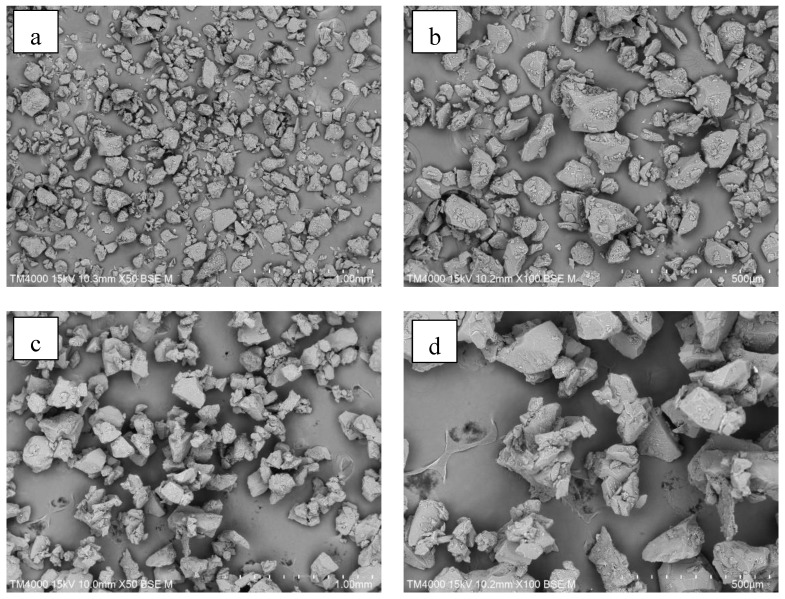

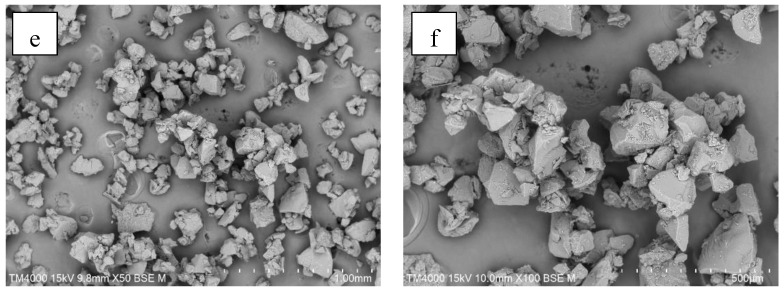
SEM images of (**a**) raw IRP (×50 magnification), (**b**) raw IRP (×100 magnification), (**c**) agglomerated IRP without bed control (×50 magnification), (**d**) agglomerated IRP without bed control (×100 magnification), (**e**) agglomerated IRP with bed control (×50 magnification), and (**f**) agglomerated IRP with bed control (×100 magnification).

Figure 8 shows SEM images for raw IRP and agglomerates with and without bed control at 50× and 100× magnification. Figure 8a,b show polysized particles of raw powder with irregular shapes but flat surfaces, which affected their cohesiveness and fluid dynamics behavior. The agglomerated particles (as seen in Figure 8c–f) exhibited a larger and looser structure, characterized by irregular shape, with a porous surface that facilitated water absorption during reconstitution. Increases in irregularity and porosity of the agglomerates were previously reported [2,11,12,18,19,20].

#### 3.3.2. Characterization of Raw and Agglomerated Instant Riceberry Powder

The physical properties, flowability, and reconstitution properties of raw and agglomerated IRP were analyzed to investigate the influence of bed stability control, as shown in Table 1. The results indicated variations in all attributes under different operations.

Inappropriate heat and mass transfer during agglomerate growth may result in high moisture content (MC) and water activity (aw) of the final product, leading to spoilage and degradation during storage [8]. The results in Table 1 show that all agglomerated IRP possessed safe levels of both critical parameters, i.e., 5.15–6.79% wb and 0.1572–0.2637 for MC and aw values, respectively. IRP agglomerated without bed control gave an MC of 5.18 ± 0.02% wb and was not significantly different when compared with the raw powder (5.15 ± 0.04% wb). The fluidized particles heated at the heat transfer zone situated just above the air distributor entered the binder spray zone, and the resulting wetted particles simultaneously collided with each other under drying, resulting in agglomerate formation [11]. Several passes through these zones provided agglomerates with a certain MC. Under suitable agglomeration conditions, the granules should have an MC similar to the initial value of the raw materials. However, after agglomeration with bed control, the moisture content of the IRP granules (6.79 ± 0.08% wb) was slightly higher than those of the raw sample and IRP obtained from no-bed-control agglomeration. This MC variation occurred because wetted or heavily enlarged granules were fluidized into the wetting zone with an adaptive flow rate during the stable bed expansion. The wetted particles received more droplets under a fixed fluidizing air temperature and binder feed rate, resulting in a lower evaporation rate for the drying mechanism. This discrepancy in MC can be resolved by controlling the binder flow rate and fluidizing air temperature or even prolonging the drying stage after spraying [8,11].

The particle size of agglomerates is commonly monitored as this is a critical output parameter representing agglomeration efficiency. In this study, IRP granule particle size was expressed in terms of D_10_, D_50_, D_90_, and span, as shown in Table 1. The sizes of raw IRP were 16.75 ± 1.22, 58.42 ± 1.61, and 148.69 ± 0.22 μm for D_10_, D_50_, and D_90_, respectively, while its span was 2.26 ± 0.08. When compared to the raw IRP, mean particle size (D_50_) of the granules increased to 237.19 ± 10.67 and 261.99 ± 18.62 μm under operations without and with bed control, respectively. Additionally, a narrower size distribution was found when using both operation modes. Enlarged particles with lower span were also found by Dacanal et al. [6], Nascimento et al. [12], Nascimento et al. [11], Atalar and Yazici [19], and Ji et al. [21]. When using pulsed fluidized-bed agglomeration with bed control, mean particle size (D_50_ = 261.99 ± 18.62 μm) improved compared to the regular process, and size distribution was narrower (span = 0.86 ± 0.04). Large particles passed into the spray zone more frequently, with segregated smaller particles also wetted and colliding with each other, resulting in larger particles with narrow size distribution.

The bulk properties of raw IRP and agglomerates were determined and presented in terms of bulk, tapped, particle density, as shown in Table 1. Agglomeration processes with both operations tested here led to a decrease in all densities of IRP. The raw IRP had bulk, tapped, particle densities of 474.40 ± 6.42, 791.02 ± 4.25, and 2367 ± 152.17 kg m^−3^, respectively. After agglomerating with no bed control, bulk, tapped, and particle densities slightly reduced to 403.91 ± 5.82, 556.33 ± 3.74, and 2219.03 ± 85.13 kg m^−3^, respectively. This observation was attributed to particle enlargement, with large intergranular spaces corresponding to increased porosities of the granules. IRP porosity increased from 66.59 ± 1.98% to 74.93 ± 0.79% and 76.73 ± 0.08% for the uncontrolled and controlled bed processes, respectively. These findings concur with those of Ji et al. [21], Atalar et al. [18], and Atalar and Yazici [19]. When considering the influence of bed control, all densities tested here were significantly lower than the raw and agglomerated powders obtained using the regular process. Larger granule size resulted in more particle passages or more residence time in the wetting zone, as a reasonable explanation of this observation when adapting the fluidizing air flow rate.

Considering its handling properties, raw IRP had a HR value of 1.667 ± 0.014, showing high cohesiveness due to its high interparticle forces [18]. As shown in Table 1, the HR value of the granules decreased to 1.377 ± 0.011 and 1.278 ± 0.007 after agglomeration without and with controlling bed expansion, respectively, indicating intermediate powder cohesiveness. Particle enlargement with higher porosity gave improved cohesiveness of all IRP agglomerates [18]. The CI value of raw IRP decreased from 40.03 ± 0.49% to 27.40 ± 0.56% and 21.78 ± 0.40% after agglomeration under fixed and adaptive fluidizing air flow rates, respectively, corresponding to the change in flowability from bad to fair. The size enlargement during agglomeration allowed the coarser IRP replacing the fine particles to retain flow stability. Improvements in the cohesiveness and flowability of the IRP sample obtained from pulsed fluidized-bed agglomeration concurred with the results of Dacanal et al. [9], Dacanal and Menegalli [8], Nascimento et al. [11], and Nascimento et al. [12,22], while the cohesiveness and flowability of IRP powders agglomerated under stable bed expansion significantly increased compared to the ordinary agglomeration mode due to more occupancy of fluidized particles inside the wetting zone, as a result of the adaptive fluidizing air flow rate.

The reconstitution of the powders was characterized in terms of their wettability and solubility, as shown in Table 1. Wetting time allowed the IRP sample to be entirely wetted and decreased from 74 ± 6.51 s to 46 ± 4.00 s and 27 ± 1.00 s when agglomerated under fixed and variable air flow rates, respectively. This improved wettability of the agglomerated IRP corresponded to increased values of dispersibility, from 28.88 ± 1.35% to 32.86 ± 2.87% and 58.09 ± 1.08%. Enlarged and more porous IRP particles had more capability to mitigate the surface tension occurring at the solid–liquid interface, resulting in a shorter time needed to sink into hot water. Thus, more particles dissolved before forming the coat wrapping the unwetted powders, with improvements in the reconstitution properties as previously reported [19,23,24].

## 4. Conclusions

This research study demonstrated the potential of pulsed fluidized-bed agglomeration as a valuable technique for improving the efficiency and quality of instant starch-based powder production. The Carr index of agglomerated instant riceberry powder (IRP) reduced from 40% to 27%, indicating improved flowability, which shifted from a bad to a fair classification. A Hausner ratio of about 1.4, classified as intermediate powder cohesiveness, also revealed reduced powder cohesiveness after agglomeration, which agreed with larger particle sizes in terms of D10, D50, and D90. Additionally, improvements in bulk and reconstitution properties were found after agglomeration. However, bed expansion captured using our image-processing method showed a progressive decrease with agglomeration time. Bed height controlled by the image-processing technique effectively enhanced hydrodynamics stability, resulting in improved agglomeration efficiency and product qualities. Consequently, pulsed fluidized-bed agglomeration with progressive airflow adjustment should be considered to achieve desirable particle characteristics and instant attributes. Future studies should examine the impacts of various pulsation frequencies, air flow rates, and binders to optimize the agglomeration process, with the aim of achieving more consistent particle properties.

## Figures and Tables

**Table 1:** Quality attributes of raw and agglomerated IRP under different operations.

Attributes	Raw Powder	Agglomerated Powder	
		No Control	Control
Moisture content (% wb)	5.15 ± 0.04 ^b^	5.18 ± 0.02 ^b^	6.79 ± 0.08 ^a^
a_w_ (-)	0.1572 ± 0.0040 ^c^	0.2068 ± 0.0092 ^b^	0.2637 ± 0.0073 ^a^
Particle size			
D_10_ (μm)	16.75 ± 1.22 ^c^	32.61 ± 4.05 ^b^	188.98 ± 19.92 ^a^
D_50_ (μm)	58.42 ± 1.61 ^c^	237.19 ± 10.67 ^b^	261.99 ± 18.62 ^a^
D_90_ (μm)	148.69 ± 0.22 ^b^	416.84 ± 12.62 ^a^	433.48 ± 26.48 ^a^
Span (-)	2.26 ± 0.08 ^a^	1.62 ± 0.03 ^b^	0.86 ± 0.04 ^c^
Bulk density (kg·m^−3^)	474.40 ± 6.42 ^a^	403.91 ± 5.82 ^b^	380.70 ± 2.70 ^c^
Tapped density (kg·m^−3^)	791.02 ± 4.25 ^a^	556.33 ± 3.74 ^b^	486.68 ± 5.94 ^c^
Particle density (kg·m^−3^)	2367.59 ± 152.17 ^a^	2219.03 ± 85.13 ^b^	2091.29 ± 17.94 ^c^
Porosity (%)	66.59 ± 1.98 ^b^	74.93 ± 0.79 ^a^	76.73 ± 0.08 ^a^
Carr index (%)	40.03 ± 0.49 ^a^	27.40 ± 0.56 ^b^	21.78 ± 0.40 ^c^
Hausner ratio (-)	1.667 ± 0.014 ^a^	1.377 ± 0.011 ^b^	1.278 ± 0.007 ^c^
Wettability (s)	74 ± 6.51 ^a^	46 ± 4.00 ^b^	27 ± 1.00 ^c^
Dispersibility	28.88 ± 1.35 ^c^	32.86 ± 2.87 ^b^	58.09 ± 1.08 ^a^

Different superscripts indicate significantly different (*p* < 0.05) means in the same row.

## Data Availability

The original contributions presented in the study are included in the article, further inquiries can be directed to the corresponding author.
